# Case of Fracture and Depression of a Part of the Frontal Bone, with an Escape of a Considerable Portion of the Brain; and from Which the Patient Completely Recovered, without Operation

**Published:** 1825-03

**Authors:** Francis Corban

**Affiliations:** Member of the Royal College of Surgeons, London.


ART. IV.—***Case of Fracture and Depression of a part of the Frontal Bone, with an Escape of a considerable portion of the Brain; and from which the Patient completely recovered, without Operation.*** By FRANCIS CORBAN, M.D. Member of the Royal College of Surgeons, London.

ON the 7th of November, 1824, I was requested to see Daniel Donovan, ætatis nine, of a robust frame, and previously unacquainted with disease, who had received a severe blow from a horse's foot about two hours before, and who was, as his father reported, just expiring. On examining the state of the general symptoms and constitutional derangement, I found the body quite cold, the lower extremities in particular; face pale and languid; pulse feeble and slow, but not intermitting; breathing rather laborious, without stertor; pupil dilated, but sensible to the natural stimulus; stomach so irritable, that it rejected every thing offered.

The boy complained of severe pain in the seat of the wound, which was covered with coagulated blood. His friends mentioned that he lost a large quantity of blood before my visit, probably from a branch of the temporal artery, which was torn. I ordered a little warm cordial to be taken in divided portions; his feet to be placed in warm water, and heat to be applied to the region of the stomach; by which he much revived in a short time. I then examined the wound, which presented the following appearance:—The frontal bone was completely denuded about three inches in length, and one or two in width, by a lacerated wound, commencing at nearly the median line of the forehead, and proceeding outwards and downwards towards the left, exposing a fracture and deep depression of a portion of bone, nearly equal in extent, which was beaten in with such violence as to rupture the dura mater, and force through it a dessert-spoonful of medullary matter. The boy, though first stupified by the blow, was now quite rational, and capable of answering any questions distinctly: in consequence of which, I simply cleaned the wound, applying a few adhesive straps to keep the lips together, and a pledget covered with mild ointment. I ordered an effervescing mixture to quiet the stomach, and a pill, consisting of calomel, jalap, and scammony, with a little ginger, to clear the bowels; a purgative enema at bed-time; and abstinence from all kinds of food, with the exception of mild diluents, &c. &c.

The remainder of the case will be given in the form of an hospital report, for the sake of brevity.

Second day.—Patient was reported to have spent a very bad night, with incoherent wandering; stomach much disturbed; frequent vomiting; much pain of head at present; pulse 110, full and strong; two dejections from the medicine; abdomen tender on pressure, particularly about the hepatic region: some medullary matter in the wound.—Let the head be shaved, and kept constantly damp with an evaporating lotion. Fiat VS. ad ℥xviij. Sumat sulp. magnesiæ ℥j. ex inf. ros. ℥v. in dies. Injiciatur enema purgans hora somni.

Third.—Raving during the night, but now perfectly sensible; bowels free; suppuration commencing in the wound, which shows no symptom of union; a portion of brain in the dressings; pulse 108; scalp hot.—Let the evaporating lotion be continued, and the enema at night.

Fourth.—Rested rather badly during the night; vomiting returned this morning; a small quantity of biscuit was retained on the stomach, but fluids were immediately rejected; lips of the wound tumid and gaping, with a sanious discharge mixed with medullary matter; pain of head severe; pulse 115, sharp; one dejection.—Let the temporal artery be opened, and twelve ounces of blood extracted. Let a soft, warm poultice be applied to the wound; the lotion continued to the head as before. Repetatur mistura ut antea, et enema hora somni si opus sit.

Fifth.—Slept for some hours since last visit; feels better in every respect; bowels open; stomach at rest.—Continuatur cataplasm emol.

Sixth.—Complains less of the head this morning than before; discharge from the wound improved in quality, but abundant. —Repetantur cataplasm et enema.

Eighth.—Some uneasiness, with feverish disturbances, during the night; sense of dull pain over the head; wound tender to the touch, and gaping; discharge thin and fetid; vomited this morning; tongue dry and foul; constipation of bowels.—Habeat Sulph. mag. ℥j. Infus. sennæ ℥viij.; sumat ʒij. ad plenam alvi solutionem. Let the lotion and poultice be repeated.

Ninth.—No sleep; sense of cord-like tightness in the fore part of the head; eyes very sensible to light, but the patient quite collected; one dejection only.—Applicatur emplastrum lyttæ nuchæ. Repetantur haustus sulph. magnesiæ et cataplasm.

Tenth.—Pain of head considerably relieved; bowels free; eyes natural; blister acted very well.—Let the ulcerated part be dressed with stimulant ointment, and the poultice continued.

Twelfth.—Continues to improve; wound suppurating kindly. —Let his diet be improved with a little broth, gruel, &c.

From this time to the twentieth, all alarming symptoms gradually subsided; wound granulated and healed, with the assistance of mild digestives only.

Twenty-second.—Let him be dismissed, cured.

*Observations.*—I visited him on the day this report was written, and had the satisfaction to find him completely restored, both in mind and body; wound healed, but a deep depression remains.

Though I am fully aware that there are many cases on record of a much more serious nature than the above, particularly in gun-shot wounds, where large quantities of the brain have sloughed away, still I am inclined to hope that this case will not be considered entirely devoid of interest, as it affords a useful lesson to surgeons who may be too hasty in the use of instruments, (as far as an individual case can go,) how powerful the resources of nature are, even under such unpromising circumstances; and how cautious they should be in adding a severe wound to that already inflicted,—I mean by the use of the trephine.

In this case, there was not only an extensive fracture, with deep depression, but such was the violence of the blow, that a quantity of the brain escaped through the ruptured dura mater; notwithstanding this, no inflammation of consequence ensued, and what did occur was kept in check by comparatively mild measures; the unfavourable symptoms on the eighth and ninth were caused by an allowance of food and wine, with which his friends rashly indulged him, and directly contrary to my directions. How far the favourable termination was promoted by the treatment adopted, I will not take on me to say; but of this I am convinced, that many well-informed surgeons in Mr. Pott's time would consider my treatment of this case rather inert, and would have been tempted to lay on the trephine, as the most effectual step towards the patient's welfare. But, as I have advanced this case in support of the *medicine expectante* in such injuries, I would wish to be clearly understood that I conceive the trephine to be a very valuable resource, where symptoms of compression, or internal accumulations of matter, exist, attended with stupor; but as, in this case, the boy was perfectly sound in intellect from the time I saw him till his recovery, I therefore ventured to abstain from it; and the result I give to the impartial reader. —

*Glandore Dispensary, Cork; Dec.* 22, 1824.

